# Apigenin Alleviates Obesity-Associated Metabolic Syndrome by Regulating the Composition of the Gut Microbiome

**DOI:** 10.3389/fmicb.2021.805827

**Published:** 2022-01-03

**Authors:** Yuan Qiao, Zhichun Zhang, Yuanyuan Zhai, Xu Yan, Wenling Zhou, Hao Liu, Lingling Guan, Liang Peng

**Affiliations:** Beijing Key Laboratory for Immune-Mediated Inflammatory Diseases, China-Japan Friendship Hospital, Institute of Medical Science, Beijing, China

**Keywords:** flavonoids, gut microbiota, fecal microbiota transplantation, obesity, lipopolysaccharides

## Abstract

The gut microbiota, often viewed as a “digestive organ,” can influence the development of obesity and related metabolic disorders. Diet is significantly important in shaping the structure and modulating the function of the gut microbiota. Apigenin (Api) widely exists in fruits and vegetables as a naturally occurring flavonoid and has anti-obesogenic, anti-inflammatory, and anti-carcinogenic properties. Its low bioavailability means it has enough time to interact with the intestine thus becomes a potential substrate for the gut intestine; thus, contributing to gut health. Here, we show that Api reduces whole-body weight, low-grade inflammation, and insulin resistance in high-fat diet (HFD)-induced obese mice. Our results reflect that Api supplementation can substantially improve intestinal dysbiosis triggered by HFD and restores gut barrier damage by alleviating metabolic endotoxemia. Augmentation of *Akkermansia* and *Incertae_Sedis* along with reduction of *Faecalibaculum* and *Dubosiella* at the genus level potentially mediated the protective effects of Api on metabolic syndrome. Furthermore, we show that the impact of Api on the reduction of body weight and the modification of gut microbiota could be transferred from Api-administered mice to HFD-feeding mice *via* horizontal fecal microbiota transplantation. Taken together, our data highlight the prebiotic role of Api and show its contribution to the restraint of gut dysbiosis and metabolic deterioration associated with obesity in mice.

## Introduction

Obesity, a metabolic disturbance, is featured by excessive accumulation of body fat due to the imbalance between energy intake and consumption (where energy intake exceeds energy expenditure; Must et al., 1999). Excessive abdominal fat increases the risk of metabolic syndrome, which is closely associated with numerous chronic diseases, such as cardiovascular disease, hypertension, dyslipidemia, non-alcoholic fatty liver disease (NAFLD), diabetes, and cancer (Despres and Lemieux, 2006). It is thus, self-evident that obesity can pose adverse effects on human health. Over the past few decades, obesity has become a serious challenge worldwide (Swinburn et al., 2011; Hoyt et al., 2014), and the urgency for effective drugs to treat this condition has increased.

The gut microbiota is the microbial community within the gastrointestinal tract. It is involved in energy homeostasis and is associated with digestion, metabolism, and immunity. The gut microbiota is often viewed as a “digestive organ” (Heintz-Buschart and Wilmes, 2018; Zmora et al., 2019). Previous studies have suggested that gut microbiota dysbiosis contributes to the obesity pathophysiology and is related to chronic metabolic diseases, such as NAFLD and diabetes (Canfora et al., 2019). A previous study showed that germ-free mice were prone to gaining weight and accumulating fat after receiving fecal transplants from high-fat diet (HFD)-induced obese mice (Ussar et al., 2015). Thus, gut microbiota dysbiosis has been confirmed to occur before the development of obesity. After the causality between gut microbiota was confirmed, subsequent studies found the impact of intestinal microbiota on metabolic disorders may be mediated through metabolites produced by microbiota like short-chain fatty acids (SCFAs), secondary bile acids (BAs), branched-chain amino acids (BCAAs), and/or proinflammatory bacteria-derived factors, such as lipopolysaccharides (LPS; Gomes et al., 2018). Dysbiosis can impair the function of the intestinal barrier to allow the passage of LPS from intestinal Gram-negative bacteria into the systemic circulation, which can stimulate Toll-like receptor 4 (TLR4)-mediated inflammation, leading to metabolic endotoxemia and insulin resistance (Plociennikowska et al., 2015). Therefore, improving the condition of the gut microbiota is a potential treatment for obesity.

In recent years, prebiotics have been confirmed to have the functions of altering the intestinal microbiota, improving intestinal barrier integrity, and decreasing endotoxemia caused by LPS, which may prevent inflammation induced by a HFD, such as isoflavones, anthocyanidins, and flavonoids especially. (Gil-Cardoso et al., 2016). Flavonoids, a type of polyphenol, are known for their anti-inflammatory, antioxidant, anti-infective, antidiabetic, and anticancer effects in humans. Its products have low bioavailability and therefore have enough time to interact with the intestine, becoming potential substrates for the gut microbiota (Gil-Cardoso et al., 2016). A naturally occurring flavonoid, apigenin (Api) widely exists in fruits and vegetables, especially in celery (Hostetler et al., 2017). It has been reported that Api possesses potent antioxidant properties and low intrinsic toxicity (Ali et al., 2016; Salehi et al., 2019). There is convincing evidence that Api treatment is effective in improving dyslipidemia and insulin resistance on HFD-induced obese mice (Lv et al., 2019). Consequently, Api has been viewed as a potential hypolipidemic agent for obese patients. However, the sophisticated mechanisms of Api’s anti-obesity effects *in vivo* are not fully understood. Our findings demonstrate that Api supplementation may represent a potential prebiotic therapy for body obesity management and its correlative metabolic derangement.

## Materials and Methods

### Animals

All animal protocols were approved and performed following the guidelines from the Animal Studies Committee of China-Japan Friendship Hospital. Male, six-week-old C57BL/6 J mice were purchased from Beijing Sibeifu Bioscience Co. Ltd. (Beijing, China) and kept in a controlled environment with available food and water. After 1 week of acclimatization, mice were `administrated with or without Api after being fed a normal diet (ND) or a HFD for 16 weeks (*n* = 6). Api was dissolved in 0.5% sodium carboxymethyl cellulose (CMC-Na) and therapeutic groups were administrated with Api at a dose of 50 mg/kg body weight *via* intragastric gavage (Lv et al., 2019). For the control experiment, mice were administrated in parallel with an identical volume of vehicle (0.5% CMC-Na). Throughout the whole study, body weight was monitored weekly and food intake was monitored. On the last day, 12 h after starvation, mice were placed in an induction chamber and anesthetized with isoflurane, then sacrificed by cardiac puncture. Blood samples from the orbital venous plexus of all mice were gathered and centrifuged at 3,000 rpm for 10 min at 4°C to obtain serum.

### Fecal Transplantation

Fecal microbiota transplantation (FMT) was managed with an established protocol (Chang et al., 2015). In brief, fecal pellets from vehicle-treated and Api-treated conventional mice were transferred to anaerobic chambers for resuspension into sterile saline (100 mg of feces/1 ml of sterile saline). The mixture was stirred vigorously and then centrifuged at 0.5 g at 4°C for 5 min. Then the supernatant was collected as transplant material. Six-week-old C57BL/6 J male receivers were fed with HFD for 4 weeks and then received fresh transplant material from HFD mice or Api-treated mice (200 μl for each mouse) *via* daily oral gavage for 25 days until being sacrificed for subsequent analysis.

### Glucose and Insulin Tolerance Tests

Mice were fasted overnight (16 h) and intraperitoneal glucose tolerance tests (GTT) were conducted with glucose at a concentration of 2 g/kg body weight. Blood glucose was determined with a Contour Glucose Meter and Contour Glucose Strips (Bayer, Berlin, Germany) using venous blood from a small tail clip. The levels of blood glucose were checked at baseline (0 min),15, 30, 60, 90, and 120 min after glucose injection. Mice were fasted for 5 h during the light cycle and insulin tolerance tests (ITT) were performed using insulin at concentration of 0.75 units/kg body weight. The method of blood glucose measurement was the same as that used for the GTT.

### Biochemical Analysis

An automatic biochemical analyzer (3,100; Hitachi, Tokyo, Japan) was used to detect the levels of serum lipids and aminotransferase. Mouse Enzyme Linked Immunosorbent Assay (ELISA) kits (R&D Systems, Minneapolis, MN, United States) were used to detect the serum inflammatory factors. Serum endotoxin was measured with a Limulus Amebocyte Lysate Kit (Cambrex BioScience, Walkersville, Maryland, United States).

### Histology and Tissue Levels of Triglyceride and Total Cholesterol

Epididymal white adipose tissue (Epi-WAT), liver, and colon (*n* = 6) were excised, weighed, and fixed in 4% paraformaldehyde for 24 h. The tissues were embedded as described previously (Cardiff et al., 2014). Liver hematoxylin and eosin (HE)-stained sections were graded using the NAFLD activity score (Kleiner et al., 2005). Liver tissues were either fixed in formalin and embedded in optimal cutting temperature medium (Sakura Finetek, Torrance, CA) or snap frozen in liquid nitrogen. Frozen liver sections were stained with oil red O (ORO) and F4/80. Immunofluorescence staining was conducted according to a standard immunofluorescent labeling protocol (Chou et al., 2020). Antibody against F4/80 was purchased from Proteintech (Wuhan, China), and all images were obtained using confocal microscopy. To measure the quantities of goblet cells, Alcian Blue periodic acid-schiff (AB-PAS) stain with colon tissue was used according to a protocol described in manufacturer’s introductions. All processed slices were observed under a light microscope. ORO and F4/80-positive areas and HE staining was assessed from at least 10 randomly selected fields per slide at ×200 magnification and analyzed using Image J software (NIH, Bethesda, MD, United States). For the triglyceride (TG) and total cholesterol (TC) analyses, the TG and TC in the livers (30–50 mg) were extracted and measured by a commercial kit (Nanjing Jiancheng Bioengineering Institute, Nanjing, China).

### Real-Time Quantitative Reverse Transcription PCR

TRIzol reagent (Invitrogen, Carlsbad, CA, United States) was used to extract the total RNA according to the manufacturer’s instructions and a previously described protocol (Jha et al., 2014). Expression data were normalized to glyceraldehyde 3-phosphate dehydrogenase (GAPDH) to adjust the variability. The primer sequences are listed in Supplementary Table S1.

### Intestinal Permeability Assay

For the intestinal permeability assay, tracer fluorescein isothiocyanate (FITC)-labeled dextran (4 kDa, Sigma, St. Louis, MO, United States) was used. After fasting for 4 h, mice were gavaged with 0.5 ml of FITC-dextran tracer at a dose of 0.4 mg/g body weight. The intestinal permeability assay was performed as described in a former study (Thevaranjan et al., 2017).

### 16S rRNA Gene Sequencing and Microbiota Analysis

Feces from each group of mice were used for the gut microbiota analysis using a method described previously (Lu et al., 2020). The sequencing of the V3–V4 regions of the 16S rRNA genes was performed as described previously using the primers 338F (5′-ACTCCTACGGGAGGCAGCAG-3′) and 806R (5′-GGACTACNNGGGTATCTAAT-3′; Munyaka et al., 2015). The microbiota data were analyzed using the Quantitative Insights Into Microbial Ecology (QIIME; Version 1.8.0) and classified into taxonomic groups using NCBI BLAST v2.6.0.

### Statistical Analysis

Unless otherwise specified, the data were processed using GraphPad Prism 8.0.2 (GraphPad Software, Inc., San Diego, CA) for statistical analysis. All biological assay data are presented as the mean ± SEM. Data sets were compared using the unpaired two-tailed Student’s *t*-test. When more than two groups were involved, data sets were compared using one-way ANOVA followed by Tukey tests for multiple-group comparisons. *p* values of <0.05 were recognized as statistically reliable. ^*^p < 0.05, ^**^p < 0.01, and ^***^p < 0.001; ^#^p < 0.05, ^##^p < 0.01, and ^###^*p* < 0.001.

## Results

### Apigenin Exhibits Robust Metabolic Protective Effects in HFD-Fed Mice

**Figure 1:**
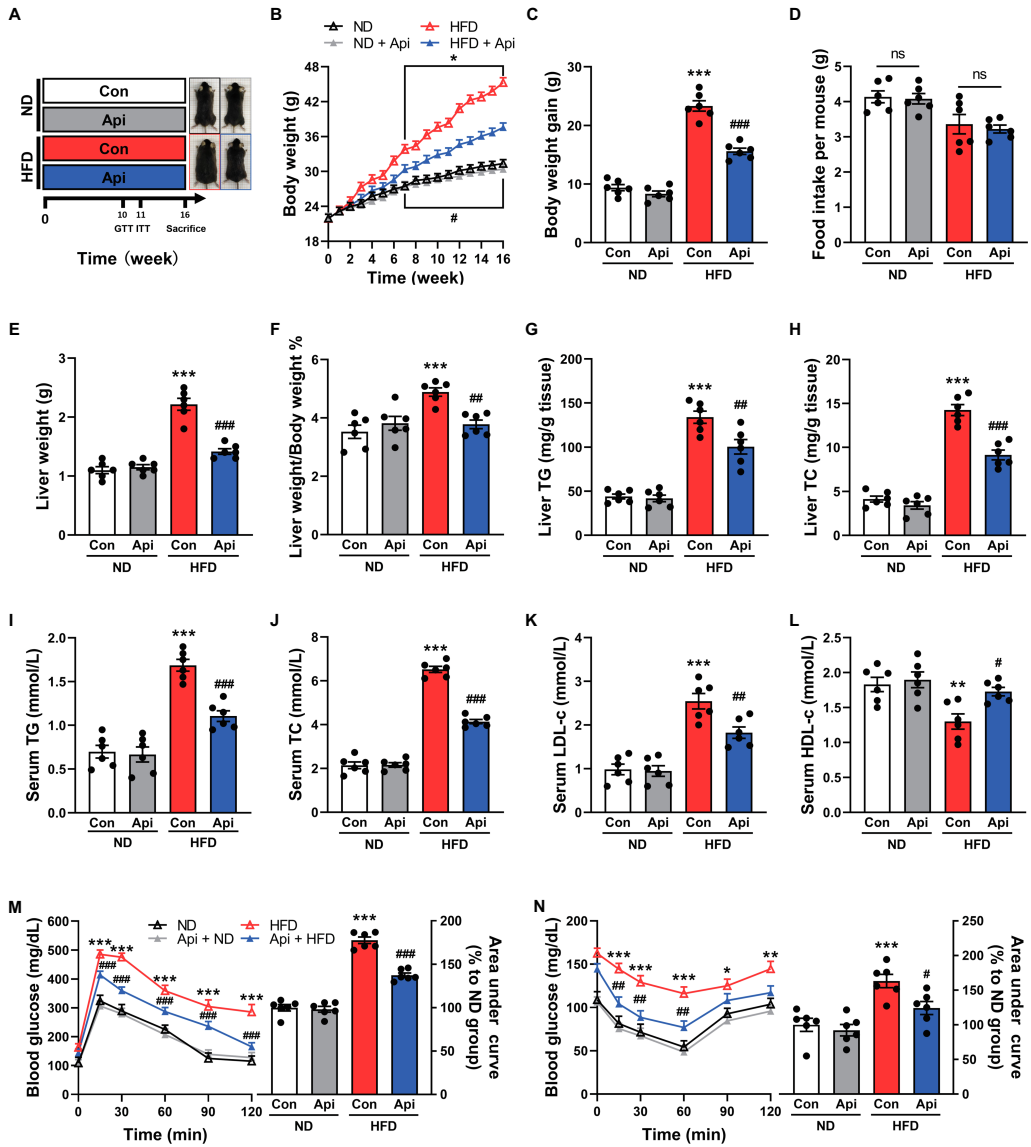
Apigenin exhibits robust metabolic protective effects in HFD-fed mice. C57BL/6 J male mice were randomly divided into four groups. **(A)** ND and HFD fed mice were treated daily with vehicle or Api (50 mg/kg) for 16 weeks by oral gavage. **(B)** Body weight for the above four groups of mice. **(C)** Body weight gain. **(D)** Average daily food intake. **(E,F)** Liver weight and Liver weight/Body weight ratio. **(G,H)** TG and TC contents in liver. Serum levels of **(I)** TG, **(J)** TC, **(K)** LDL-c, and **(L)** HDL-c were determined. **(M)** GTT results and area under the curve of GTT. **(N)** ITT results and area under the curve of ITT. Error bars are expressed as means ± SEM (*n* = 6). Statistical significance was determined by one-way ANOVA with Tukey tests for multiple-group comparisons. ^*^p < 0.05, ^**^p < 0.01, and ^***^p < 0.001 vs. ND ^#^*p* < 0.05 ^##^*p* < 0.01, and ^###^*p* < 0.001 vs. HFD.

To investigate the potential therapeutic effect of Api on obesity and its associated complications, we fed mice with a HFD for 16 weeks (Figure 1A) and observed that the body weight and body weight gain were markedly elevated compared with the ND feeding (Figures 1B,C). Compellingly, the supplementation of Api significantly abolished the obesity traits without affecting energy intake (Figure 1D), indicating that this treatment did not regulate appetite. The effect on weight gain induced by Api was particularly obvious in the liver (Figures 1E,F) and Epi-WAT (Supplementary Figures S1A,B). Moreover, Api administration reduced hepatic TG and TC accumulation in HFD-fed mice (Figures 1G,H). We also measured the fat accumulation *via* serum lipids. The serum levels of TG, TC, and low-density lipoprotein-cholesterol (LDL-c) were found to be lower in Api-treated mice compared to HFD-fed controls, while the serum level of high-density lipoprotein-cholesterol (HDL-c) was elevated in Api-treated mice (Figures 1I–L). Next, we investigated the effect of Api on insulin sensitivity using GTT and ITT. As shown in Figures 1M,N, the administration of Api markedly improved glucose intolerance and enhanced insulin sensitivity after HFD feeding. These findings indicate that Api may offer a metabolic protective function in HFD-fed mice by alleviating obesity and insulin resistance.

### Apigenin Ameliorates HFD-Induced Systemic Inflammation, Steatohepatitis, and Adipose Tissue Inflammation in Mice

**Figure 2:**
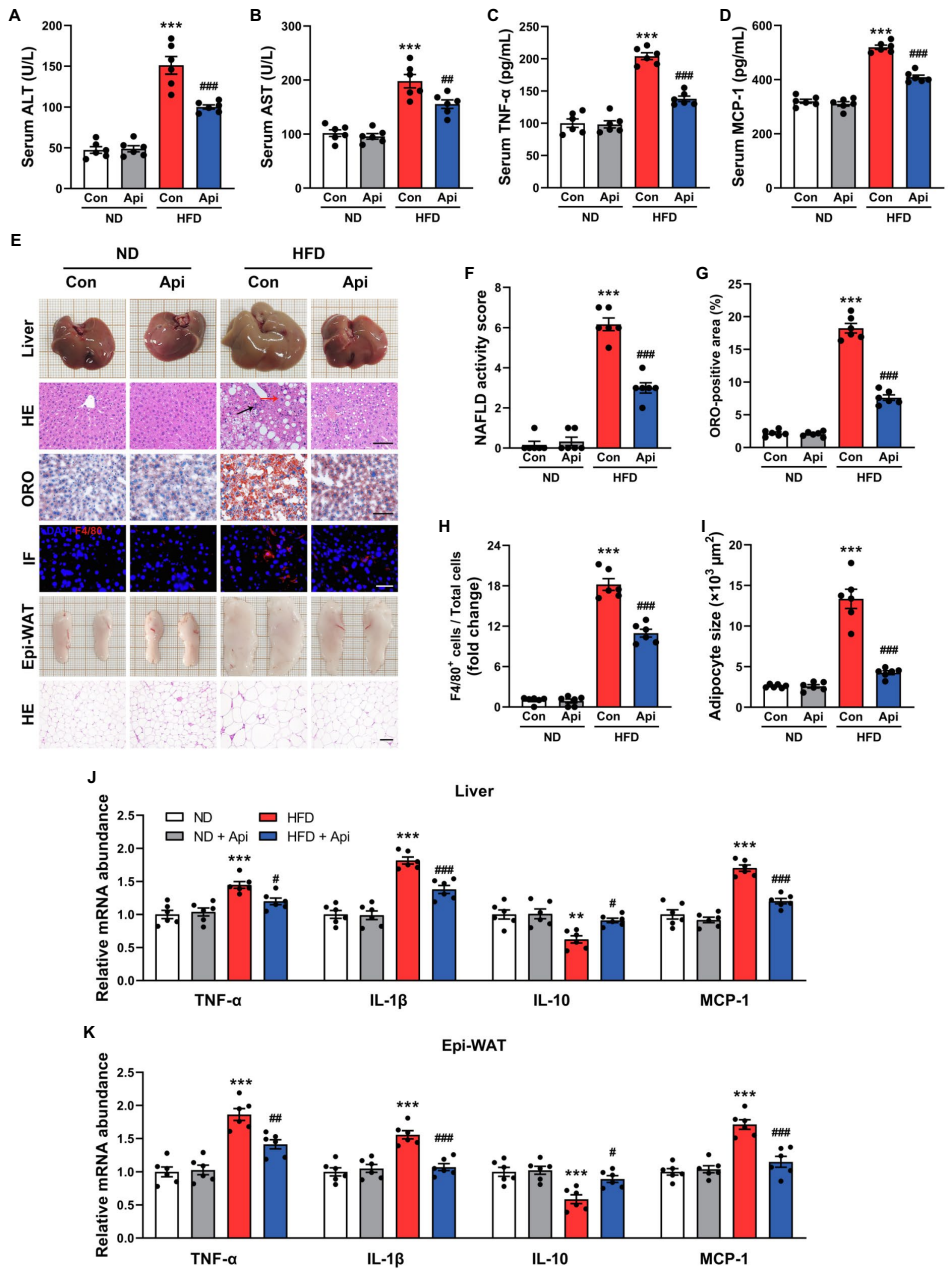
Apigenin ameliorates HFD-induced systemic inflammation, steatohepatitis, and adipose tissue inflammation in mice. **(A,B)** Serum levels of ALT and AST. **(C,D)** Serum levels of TNF-α and MCP-1. **(E)** The gross appearance of the liver. Liver lipid accumulation was assessed by HE and ORO staining, scale bar, FIGURE 2 | 100 μm. Immunofluorescence staining for F4/80 in liver tissue, scale bar, 50 μm. The gross appearance of the Epi-WAT. HE staining of Epi-WAT, scale bar, 100 μm. **(F)** NAFLD activity score. **(G)** Quantification of ORO staining. **(H)** Quantification of F4/80^+^ staining. **(I)** Quantification of adipocyte size. **(J)** Relative expression of TNF-α, IL-1β, IL-10, and MCP-1 in hepatic was assessed using qRT-PCR and in comparison with the ND group. (K) Relative expression of TNF-α, IL-1β, IL-10, and MCP-1 in Epi-WAT was assessed using qRT-PCR and in comparison with the ND group. Error bars are expressed as means ± SEM (*n* = 6). Statistical significance was determined by one-way ANOVA with Tukey tests for multiple-group comparisons. ^*^p < 0.05, ^**^p < 0.01, and ^***^p < 0.001 vs. ND; ^#^*p* < 0.05 ^##^*p* < 0.01, and ^###^*p* < 0.001 vs. HFD.

Obesity is characterized by chronic low-grade inflammation, which is a critical factor in the pathogenesis of NAFLD (Osborn and Olefsky, 2012). Therefore, we measured the inflammation-related indexes after 16 weeks of HFD-feeding in mice that had received Api treatment and in control mice. As shown in Figures 2A,B, Api supplementation restored the liver injury triggered by HFD feeding, revealed by decreased serum levels of alanine aminotransferase (ALT) and aspartate aminotransferase (AST). Moreover, we found that the serum level increments of proinflammatory cytokines, including tumor necrosis factor-α (TNF-α) and monocyte chemoattractant protein-1 (MCP-1), were visually reversed by Api supplementation (Figures 2C,D). In addition, morphological observations in the liver (Figure 2E) exhibited that Api visually inhibited the fat accumulation in the liver induced by HFD feeding, which was confirmed by hepatic HE and ORO staining (Figure 2E). As shown by the NAFLD activity scores in Figure 2F, under hepatic HE staining, we observed ballooned hepatocytes (red arrows), steatosis, and lobular inflammation (black arrows) in HFD-fed mice, indicating the formation of non-alcoholic steatohepatitis; however, Api supplementation blocked the development. In Figures 2E,G, large lipid droplets could be found in liver from HFD-fed mice after ORO staining, but this lipid accumulation was hindered by Api. Coupling with that, the immunofluorescent staining of F4/80 (Figure 2E), a specific biomarker of Kupffer cells, exhibited less F4/80-positive cell number in the liver of Api-treated mice after HFD feeding compared with HFD-fed mice who did not receive Api treatment (Figure 2H). HE staining of the Epi-WAT (Figure 2E) revealed that Api supplementation significantly inhibited the enlargement of adipocytes in mice (Figure 2I). At the transcriptional level, we assessed the messenger RNA (mRNA) expressions of the proinflammatory cytokines, TNF-α, MCP-1, and interleukin 1β (IL-1β), and the anti-inflammatory cytokine, interleukin 10 (IL-10), in the liver (Figure 2J), and in the Epi-WAT (Figure 2K). Intriguingly, we observed that the levels of TNF-α, MCP-1, and IL-1β were significantly lower (*p* < 0.001), while the level of IL-10 was increased in Api-treated mice compared with control mice. Taken together, these findings shed light on the fact that Api ameliorates inflammation in HFD-fed mice.

### Apigenin Reverses Intestinal Barrier Damage Induced by HFD Feeding

**Figure 3:**
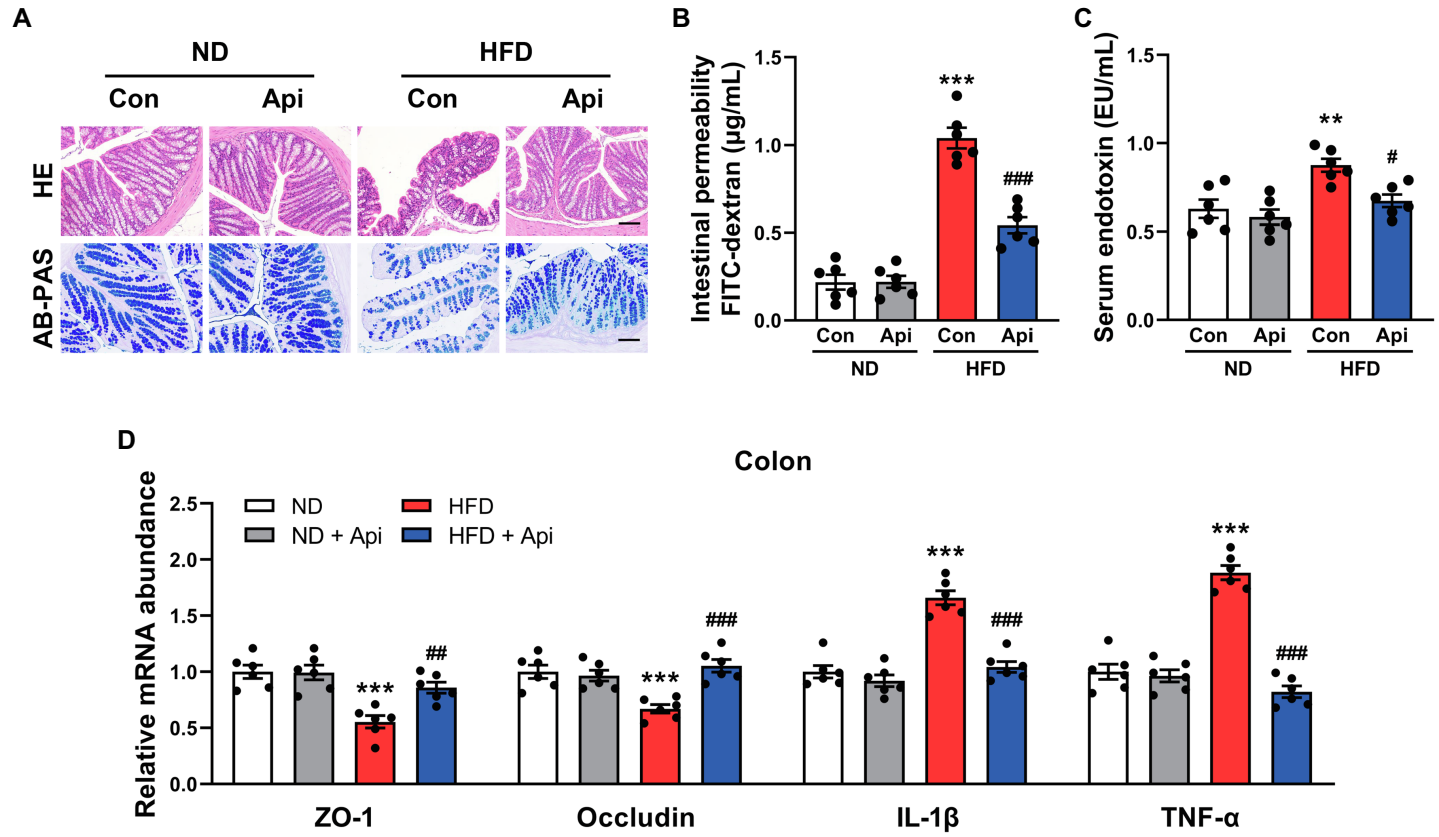
Apigenin reverses intestinal barrier damage induced by HFD feeding. **(A)** HE staining and AB-PAS staining of sections from the colon. Scale bar, 100 μm. **(B)** The intestinal permeability. **(C)** Serum levels of endotoxin. **(D)** Relative expression of ZO-1, occludin, IL-1β, and TNF-α in the colon was assessed using qRT-PCR and in comparison with the ND group. Error bars are expressed as means ± SEM (*n* = 6). Statistical significance was determined by one-way ANOVA with Tukey tests for multiple-group comparisons. ^*^p < 0.05, ^**^p < 0.01, and ^***^p < 0.001 vs. ND; ^#^p < 0.05 ^##^p < 0.01, and ^###^p < 0.001 vs. HFD.

As suggested by previous studies, HFD feeding contributed to an increase in intestinal permeability and promoted the circulating LPS levels that give rise to the development of metabolic endotoxemia and chronic low-grade inflammation (Cani et al., 2007; Cani et al., 2008). According to histologic analysis and real-time quantitative reverse transcription PCR (qRT-PCR) assay, we found that Api supplementation could restore the damaged enteric barrier in HFD-fed mice. HE staining of the colon (Figure 3A) showed that the intestinal structure was severely damaged in HFD feeding compared with ND feeding, which was partially reversed by Api supplementation. AB-PAS staining of the colon (Figure 3A) demonstrated that the goblet cell quantity was obviously decreased in HFD-fed mice, while the supplementation of Api restored the damage induced by HFD. Concomitantly, Api supplementation markedly reduced the intestinal permeability (Figure 3B) as well as the levels of serum endotoxin (Figure 3C) in obese mice. These findings were accompanied by enhanced mRNA expressions of zonula occludens-1 (ZO-1) and occludin (tight-junction genes; Figure 3D). The mRNA expression level of the proinflammatory cytokines, IL-1β and TNF-α, were also significantly reduced by Api (Figure 3D). These results confirm that Api supplementation is of great significance in maintaining intestinal epithelium integrity.

### Apigenin Administration Eliminates Gut Microbiota Dysbiosis Induced by HFD

**Figure 4:**
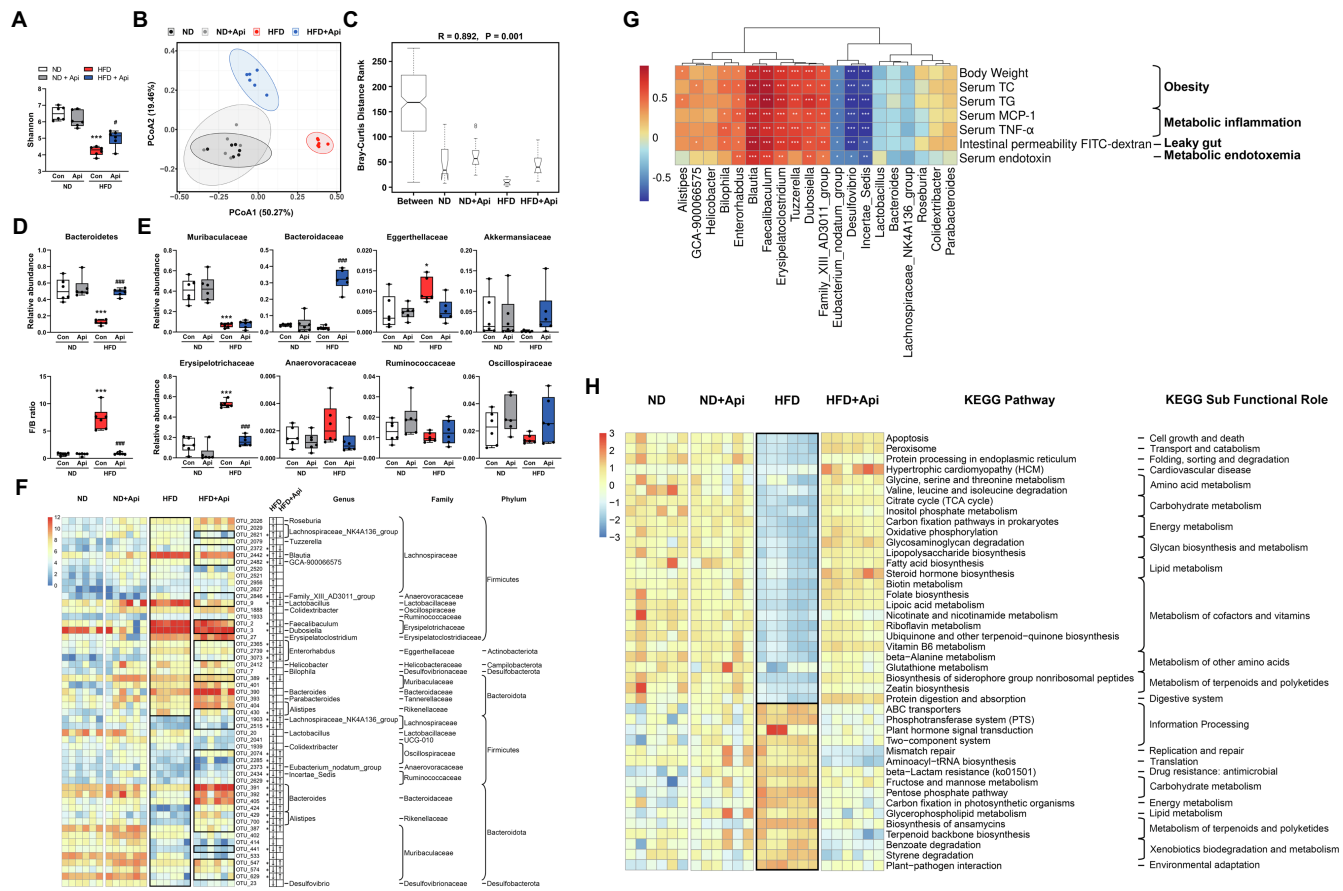
Apigenin administration eliminates gut microbiota dysbiosis induced by HFD. **(A)** Shannon index. **(B)** PCoA plot of weighted UniFrac distance. **(C)** ANOSIM analysis. R, ANOSIM test statistic. **(D)** Relative abundances of dominant bacterial at the phylum level with significant differences between groups. **(E)** Relative abundances of dominant bacterial at the family level with significant differences between groups. The first two taxa and last four taxa belong to the phyla of Bacteroidetes and Firmicutes, respectively. **(F)** Heat map showing the abundance of 54 OTUs significantly changed by HFD based on core OTUs (*p* < 0.05). Bacteria taxa information (genus, family, and phylum) of these OTUs is also shown. In the heat map, the black boxes highlight the OTUs that were significantly altered by HFD and those that were significantly reversed by Api interventions. Arrows (“↑” and “↓”) in the black boxes represent OTUs significantly altered by the HFD and reversed by the Api. ^*^OTUs whose abundance in ND feeding mice was altered by HFD and reversed by Api treatments. **(G)** Spearman correlation analysis between the 20 identified bacterial genera and obesity traits. False discovery rate correction for multiple testing was used. **(H)** The relative numbers of 42 important KEGG pathways that were significantly altered by HFD and reversed by Api treatments, their KEGG sub-functional role are depicted on the right. In the heat map, the black boxes highlight the pathways that were significantly altered by HFD. Error bars are expressed as means ± SEM (*n* = 6). Statistical significance was determined by one-way ANOVA with Tukey tests for multiple-group comparisons. ^*^p < 0.05, ^**^p < 0.01, and ^***^p < 0.001 vs. ND; ^#^*p* < 0.05 ^##^*p* < 0.01, and ^###^*p* < 0.001 vs. HFD.

The low bioavailability of Api means it has enough time to interact with the intestinal and thus may regulate the gut microbiota. To assess the mechanism of Api’s effects on the intestinal microbiota structure, high-throughput sequencing of the 16S rRNA gene, based on the hypervariable region V3–V4, was conducted to evaluate the gut microbiota composition. In the present study, the sequencing generated an average of 89,362 raw reads for each sample. The average of 84,097 clean tags were obtained after removing the low-quality sequences, and these tags were subsequently subjected to the following analysis and clustered into operational taxonomic units (OTUs). Alpha-diversity analysis, represented by the Shannon index, showed that apigenin administration markedly restored the richness and diversity of the gut microbiota that had been changed by HFD feeding at the OTU level (Figure 4A). Furthermore, we observed a distinct cluster of microbial structure for each group in the beta-diversity analysis, which was conducted *via* OTU distance-based weighted UniFrac-based principal coordinates analysis (PCoA; Figure 4B). PCoA revealed that ND feeding and Api supplementation after ND feeding showed a similar structure. 16 weeks of HFD feeding drastically changed the microbiota composition while the Api-treated group restored it to a large extent. The non-parametric analysis of similarities (ANOSIM) test was carried out to assess if the inter-group distinction was more obvious than intra-group gap thus to verify the grouping effectiveness. The ANOSIM analyses detected that the inter-group differences in community composition and abundance of the four groups were more pronounced than those within group, where *p* < 0.05 indicated reliability of the result (*R* = 0.892, *p* = 0.001; Figure 4C).

To investigate the specific variations in bacterial profiles, we evaluated the differences in the relative abundances of OTUs and taxonomic groups between the groups of mice. Compared to ND-fed mice, HFD-fed mice showed an apparent diminution in the relative abundance of Bacteroidetes and an increment of Firmicutes/Bacteroidetes (F/B) ratio, while Api supplementation protected against this impact to an extensive degree at the phylum level (Figure 4D). As for the family level, there was a diminution in the relative abundance of Muribaculaceae in HFD-fed mice, which interpreted the decrease in Bacteroidetes with HFD feeding, did not be restored by Api supplementation (Figure 4E). Compared to the other three groups, Bacteroidaceae was significantly increased by Api supplementation after HFD feeding (Figure 4E). Similarly, the increase in Firmicutes abundance induced by HFD feeding was characterized by a remarkable increase in the relative abundances of Erysipelotrichaceae and Anaerovoracaceae. Api supplementation markedly reversed the abundance of Erysipelotrichaceae (Figure 4E). Furthermore, mice feeding with HFD were found to have significantly elevated relative abundances of Eggerthellaceae yet decreased relative abundances of Akkermansiaceae, Ruminococcaceae, and Oscillospiraceae. However, Api intervention reversed their relative abundances to some extent (Figure 4E).

The current 24 data sets yielded a core microbiome (core OTUs across 100% of samples) composed of 83 species, while 54 species-level OTUs were visually transformed by HFD feeding (*p* < 0.05). HFD feeding was correlated with a distinct increment in the abundance of 29 OTUs including 17 genera, 14 families, and 5 phyla, 13 of which were transformed by Api supplementation, and with a distinct reduction in 25 OTUs including 8 genera, 10 families, and 3 phyla, 18 of which were restored after oral-administrated Api (Figure 4F). Taken together, the dietary Api administration remarkably transformed the gut microbiota composition altered by HFD feeding at the levels of microbial richness, taxonomic composition, and OTUs, elucidating the ability of Api administration to reprogram the gut microbiota.

To comprehensively analyze the relationship between obesity-related indexes and the 20 identified bacterial strains changed by HFD feeding at the genus level, Spearman correlation analysis was performed. Eight bacterial strains (*Bilophila, Enterorhabdus, Blautia, Faecalibaculum, Erysipelatoclostridium, Tuzzerella, Dubosiella*, and *Family_XIII_AD3011_group*) were found to be strongly positively correlated with the body weight, serum lipids, serum inflammatory factors, intestinal permeability FITC−dextran, and serum endotoxin, while three bacterial strains (*Eubacterium_nodatum_group, Desulfovibrio*, and *Incertae_Sedis*) were found to be strongly negatively correlated with these indexes (Figure 4G). These results suggest that these bacteria may be important bacterial strains in the development of obesity and the prevention of obesity, respectively.

To further elucidate the potential role of the intestinal microbiota changed by HFD and Api interventions, we used Phylogenetic Investigation of Communities by Reconstruction of Unobserved States 2 (PICRUSt2) analysis. According to the results, 42 Kyoto Encyclopedia of Genes and Genomes (KEGG) pathways were significantly modified by the HFD and synchronously restored by dietary Api interventions based on the Wilcoxon test (*p* < 0.05; Figure 4H). In contrast to the ND-fed group, the HFD-fed group displayed 26 markedly downregulated pathways and 16 upregulated pathways that were restored by Api administration (Figure 4H). In these identified pathways, HFD feeding significantly elevated mismatch repair and aminoacyl-tRNA biosynthesis, but significantly decreased apoptosis, oxidative phosphorylation, and lipopolysaccharide biosynthesis. All of these transformations were restored by Api administration (Figure 4H). Taken together, these findings reveal the capabilities of dietary Api intervention to regulate the metabolic pathways and functional activities of the gut microbiota.

### The Metabolic Protection of Apigenin Can Be Transferred by Fecal Transplantation

**Figure 5:**
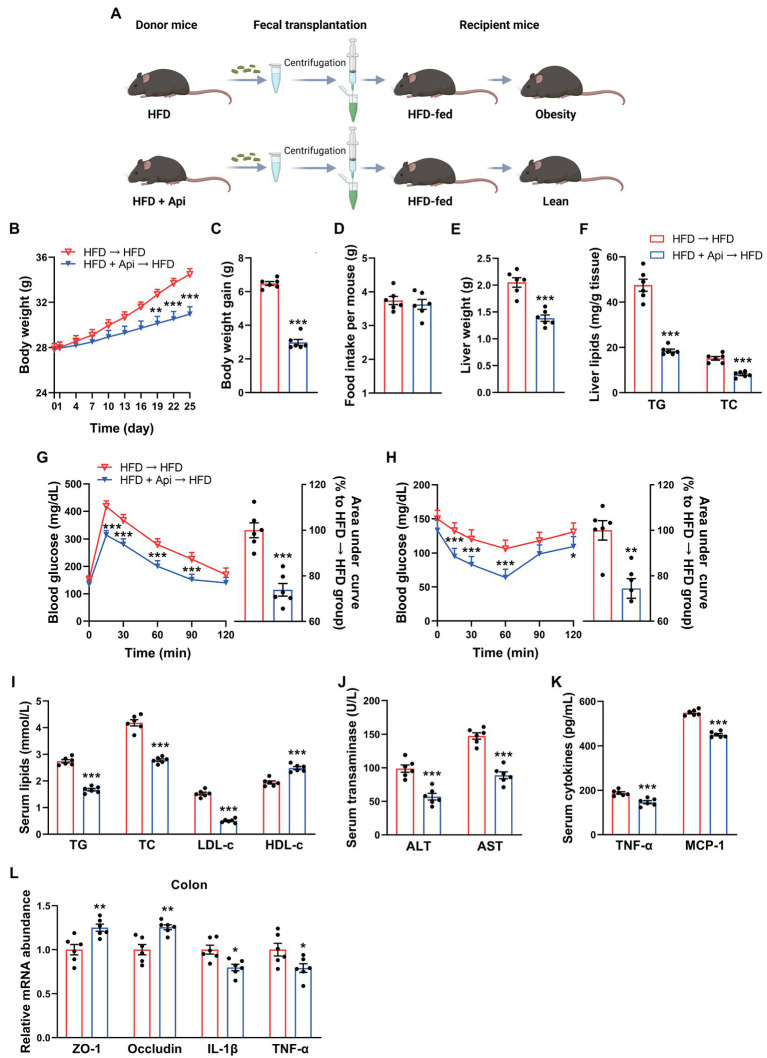
The metabolic protection of apigenin can be transferred by fecal transplantation. **(A)** Study design of fecal transplant experiment. Created with BioRender.com. **(B)** Body weight. **(C)** Body weight gain. **(D)** Average daily food intake. **(E)** Liver weight. **(F)** TG and TC contents in liver. **(G)** GTT results and area under the curve of GTT. **(H)** ITT results and area under the curve of ITT. **(I)** Serum levels of TG, TC, LDL-c, and HDL-c. **(J)** Serum levels of ALT and AST. **(K)** Serum levels of TNF-α and MCP-1. **(L)** Relative expression of ZO-1, occludin, IL-1β, and TNF-α in the colon was assessed using qRT-PCR. Error bars are expressed as means ± SEM (*n* = 6). Statistical significance was determined by unpaired two-tailed Student’s *t*-test between two groups. ^*^p < 0.05, ^**^p < 0.01, and ^***^p < 0.001 vs. HFD → HFD.

To clarify the metabolic protective roles of Api that are promoted by the intestinal microbiota, we experimented with FMT. Mice who received horizontal fecal transfer from solvent (0.5% CMC-Na)-treated HFD-fed mice were denoted as HFD receivers (HFD → HFD). Mice who received horizontal fecal transfer from Api-treated mice were denoted as Api receivers (HFD + Api → HFD). Fecal samples collected from HFD-fed mice with or without Api supplementation were transplanted into antibiotic-treated obese mice (*n* = 6; Figure 5A). Then, recipient mice were further fed with HFD for 25 days. As shown in Figures 5B–D, despite the identical food intake for both groups, the body weight and body weight gain were visually lower in the Api receivers than in the HFD receivers. The liver weight and the hepatic TG and TC content were markedly reduced in Api receivers compared with HFD receivers (Figures 5E,F). Meanwhile, Api receivers showed markedly mitigated HFD-induced blood glucose increase accompanied by improvement in insulin resistance (Figures 5G,H), ameliorated dyslipidemia (Figure 5I), restoration of liver injury (Figure 5J), and improved inflammatory cytokines in serum (Figure 5K). These results suggest that Api-treated microbiota could attenuate HFD-induced obesity and the related metabolic disorders. The amelioration effects of Api could be attributed to its modulation of the gut microbiota. Furthermore, compared with HFD receivers, the altered mRNA expression of ZO-1, occludin, IL-1β, and TNF-α were observed to be restored in Api receivers, suggesting improved intestinal barrier integrity (Figure 5L). Taken together, these results demonstrate the possible ameliorating role of Api in intestinal microbiota regulation.

### Apigenin FMT Modulates Gut Microbiota Dysbiosis Triggered by HFD

**Figure 6:**
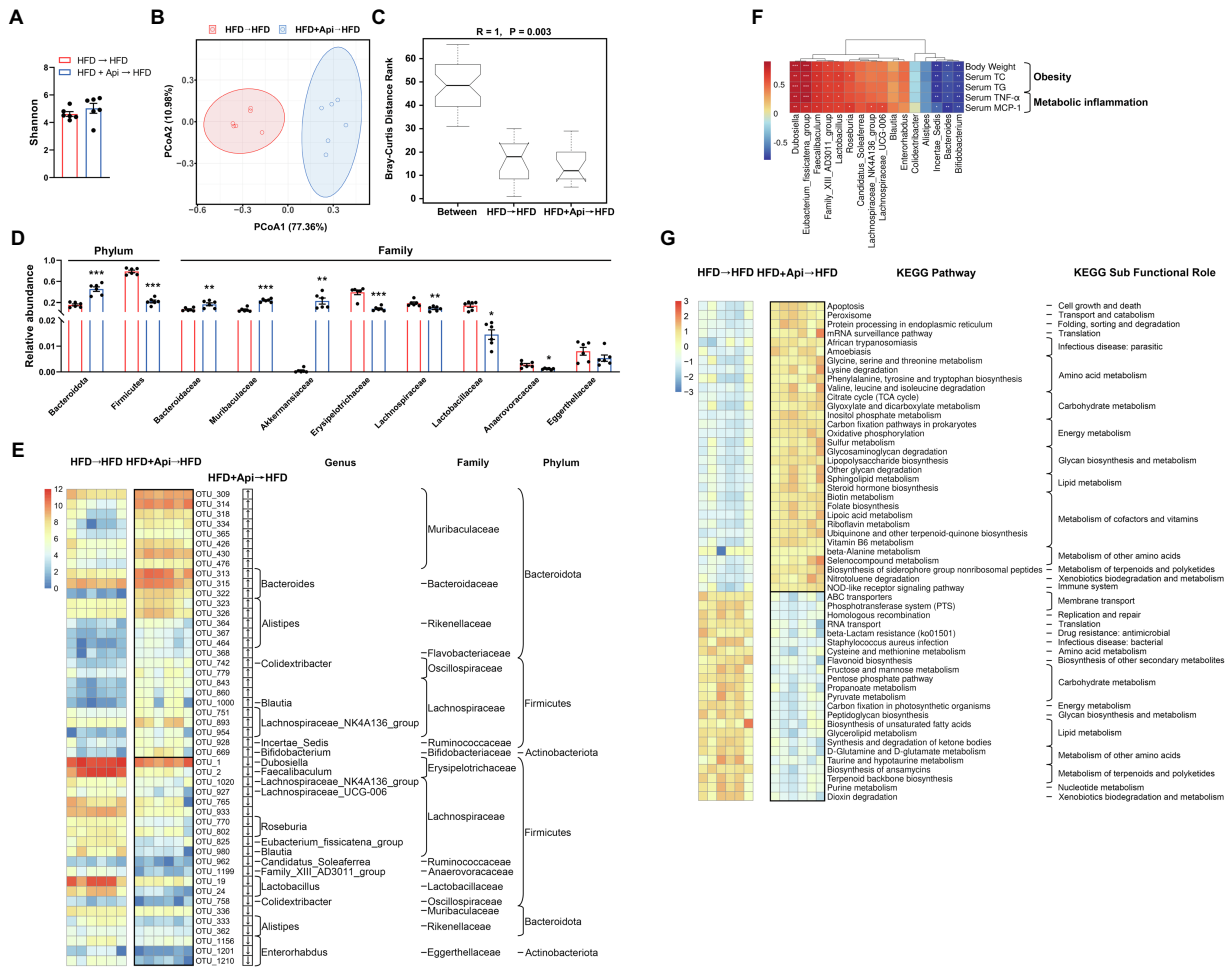
Apigenin FMT modulates gut microbiota dysbiosis triggered by HFD. **(A)** Shannon index. **(B)** PCoA plot of weighted UniFrac distance. **(C)** ANOSIM analysis. **(D)** Relative abundances of dominant bacterial at the phylum and family level with significant differences between groups. First two taxa and last eight taxa belong to the phylum and family, respectively. **(E)** The heat map shows abundance of 48 OTUs that have significant differences between the two groups based on core OTUs (*p* < 0.05). Bacteria taxa information (genus, family, and phylum) of these OTUs is also shown. In the heat map, the black boxes highlight the OTUs that were significantly altered in Api receivers. Arrows (“↑” and “↓”) in the black boxes represent OTUs that were changed by Api receivers compared with HFD receivers. **(F)** Spearman correlation analysis between the 16 identified bacterial genera and obesity traits. False discovery rate correction for multiple testing was used. **(G)** The relative numbers of 55 important KEGG pathways that have significant differences between the two groups, their KEGG sub-functional role are depicted on the right. In the heat map, the black boxes highlight the pathways that were significantly altered in Api receivers. Error bars are expressed as means ± SEM (*n* = 6). Statistical significance was determined by unpaired two-tailed Student’s *t*-test between two groups. ^*^p < 0.05, ^**^p < 0.01, and ^***^p < 0.001 vs. HFD → HFD.

In order to further confirm the anti-obesity roles of Api mediated by the gut microbiota, we examined the intestinal microbiota profiles of recipient mice with HFD feeding following fecal transfer from Api-treated mice. We adopted high-throughput sequencing of the bacterial 16S rRNA gene based on the hypervariable region, V3–V4, to evaluate the impact after receipt of an Api-treated fecal transplant. In a separate sequencing analysis, we generated an average of 71,626 raw reads for each sample (*n* = 6). The average of 68,787 clean tags were gained after removing the low-quality sequences, and these tags were subsequently subjected to the following analysis and clustered into OTUs. The Shannon index indicated that Api receivers had restored gut microbiota richness and diversity on the OTU level, but the change did not reach a statistical difference (Figure 6A). In comparison with HFD receivers, Api receivers showed distinct microbial structures *via* beta-diversity analysis, exhibited by the OTU distance-based weighted UniFrac-based PCoA (Figure 6B). The ANOSIM analyses detected that the inter-group differences in community composition and abundance between HFD receivers and Api receivers were more distinguishing than the intra-group gap and *p* value below 0.05 also presents its validity. (*R* = 1, *p* = 0.003; Figure 6C).

To further illustrate the specific variations in the bacterial profiles, we evaluated the differences in the relative abundances of OTUs and taxonomic groups between the two groups. Compared to HFD receivers, Api receivers presented a remarkable increment in the relative abundance of Bacteroidetes and a decrease in Firmicutes, indicating that Api supplementation protected against this effect at the phylum level (Figure 6D). As for the family level, the diminution in the relative abundances of Bacteroidaceae and Muribaculaceae with HFD feeding, which interpreted the decrease in Bacteroidetes with HFD feeding, was significantly restored in Api receivers (Figure 6D). Similarly, the increase in Firmicutes in HFD receivers was characterized by a distinct increment in the relative abundances of Erysipelotrichaceae, Lachnospiraceae, Lactobacillaceae, and Anaerovoracaceae, which were significantly restored in Api receivers (Figure 6D). Furthermore, HFD receivers had a significantly elevated relative abundance of Eggerthellaceae yet decreased that of Akkermansiaceae. The Api receivers had markedly reversed relative abundances of Akkermansiaceae (Figure 6D).

The current 12 data sets yielded a core microbiome (core OTUs across 100% of samples) composed of 92 species, while 48 species-level OTUs differed considerably between the two groups (*p* < 0.05). Compared with HFD receivers, Api receivers were correlated with a distinct increment in the abundance of 27 OTUs including 7 genera, 8 families, 3 phyla, and with a distinct reduction in 21 OTUs including 13 genera, 9 families, and 3 phyla (Figure 6E). Taken together, the results at the levels of microbial richness, taxonomic composition, and OTUs indicate that the anti-obesity effect of dietary Api administrations is partly mediated by the gut microbiota.

We also performed Spearman correlation analysis to investigate the relationships between obesity-related indexes and the identified 16 bacterial strains that were changed in Api receivers at the genus level. Five bacterial strains (*Dubosiella*, *Eubacterium_fissicatena_group*, *Faecalibaculum*, *Family_XIII_AD3011_group*, and *Lactobacillus*) were found to be strongly positively correlated with the body weight, serum lipids, and serum inflammatory factors, while three bacterial strains (*Incertae_Sedis*, *Bacteroides*, and *Bifidobacterium*) were found to be strongly negatively correlated with these indexes (Figure 6F). These results suggest that these bacteria may be the significant bacterial strains for the development of obesity and prevention of obesity, respectively.

Moreover, according to the results of the PICRUSt2 analysis, 55 KEGG pathways showed obvious differences between HFD receivers and Api receivers based on the Wilcoxon test (*p* < 0.05). Compared with HFD receivers, Api receivers had 32 markedly upregulated pathways and 23 downregulated pathways. In these identified pathways, Api receivers showed significantly elevated pathways for apoptosis, oxidative phosphorylation, and lipopolysaccharide biosynthesis (*p* < 0.05), but significantly downregulated pathways for homologous recombination and RNA transport (*p* < 0.05; Figure 6G). Taken together, these results indicate that Api intervention may play a vital role in regulating the metabolic pathways and functional activities of the gut microbiota.

## Discussion

**Figure 7:**
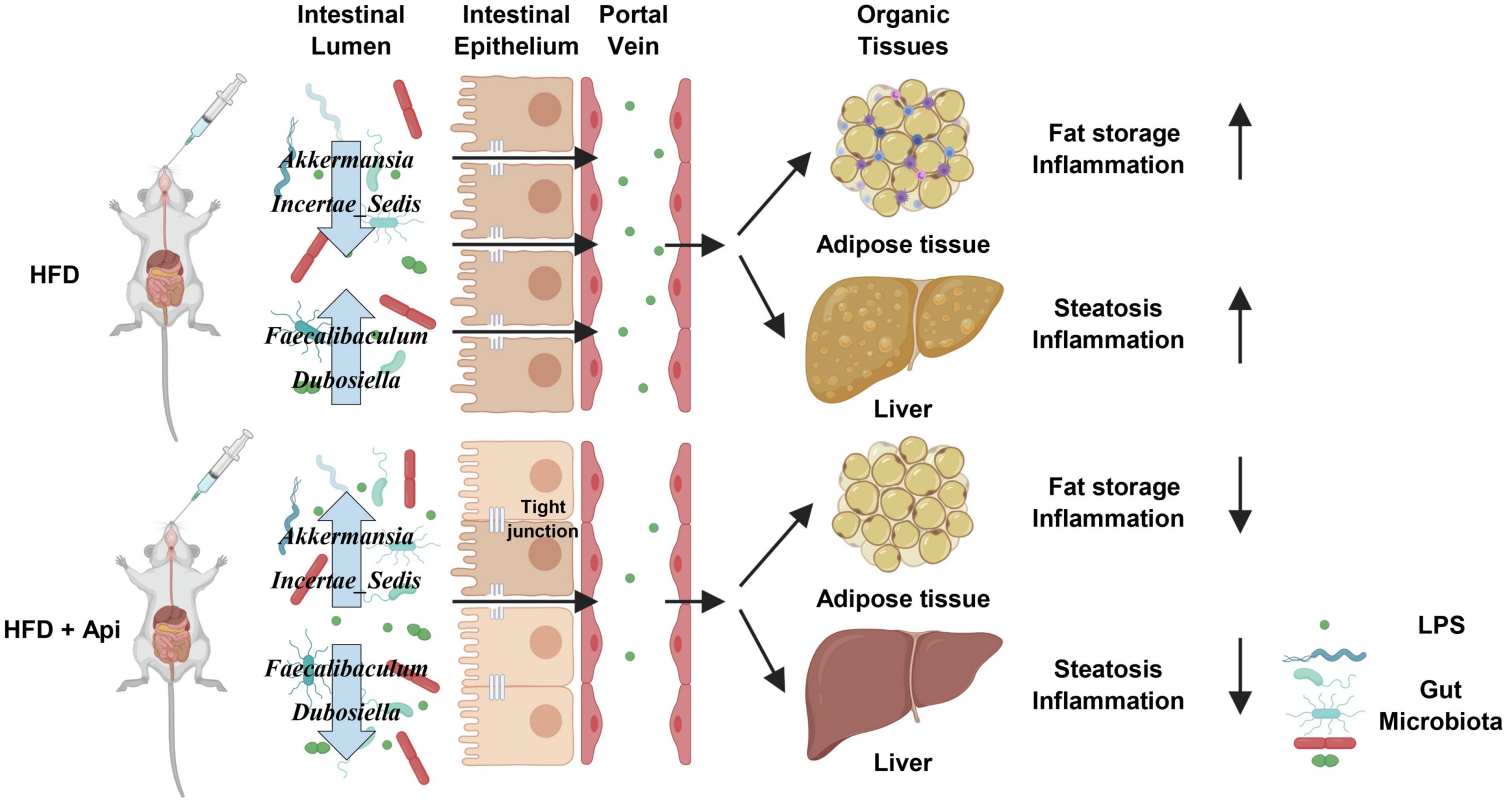
Proposed model for the metabolic protective effects of apigenin in HFD-fed mice. Api supplementations produce many beneficial effects on HFD-fed mice, including increasing tight junction expression while decreasing gut permeability, serum endotoxin levels, and inflammation. Api supplementations also reduce adipocyte inflammation. Created with BioRender.com.

Intensive investigations have proved that gut microbiota dysbiosis is closely associated with dietary habits and can lead to the development of a metabolic syndrome caused by obesity (Miele et al., 2009; Marchesi et al., 2016). Further research has revealed that the potential mechanism for the influence of the intestinal microbiota on obesity-related metabolic disorders may include energy extraction capacity from daily food, influence of the integrity of the intestinal barrier. Consequently, facilitating LPS translocation into the systemic circulation, modification of the immune system, and production of bacterial metabolites such as BAs, SCFAs, BCAAs, and trimethyl-amine-N-oxide, etc. (Rastelli et al., 2018; Sun et al., 2018). Based on these studies, there remains a great demand for the development of safe and effective microbiota-targeted treatments for metabolic syndrome. An increasing amount of evidence has shown that dietary flavonoids, such as camu, citrus, myricetin, and curcumin (Anhe et al., 2019; Zeng et al., 2020; Li et al., 2021; Sun et al., 2021), are not totally absorbed by the intestinal tract and can be metabolized by the intestinal microbiota, indicating that flavonoids may dominate in the maintenance of gut health (Gil-Cardoso et al., 2016). Api, one of the naturally occurring flavonoids that exists in daily fruits and vegetables, like plant-derived beverages, grapefruit, celery, parsley, and onions, has aroused research interests in recent years for health promotion benefiting from its negligible intrinsic toxicity, antioxidant, and anticancer properties (Shao et al., 2013; Feng et al., 2017). As shown in Figure 7, our study demonstrated that orally administrated Api can modulate the composition of the gut microbiota, promote intestinal barrier integrity, and reduce endotoxemia caused by LPS, thus protecting mice from obesity-induced inflammation and metabolic disturbance. We also reveal that the Api-altered gut microbiota performs as a causal role in the improvement of metabolic disturbance by FMT.

At the taxonomic level, as suggested by previous studies, obesity and metabolic disorders have been related to poor gut bacterial richness (Cotillard et al., 2013; Le Chatelier et al., 2013). Similarly, our results showed that the gut microbiota of HFD-fed mice had significantly lower alpha-diversity than that of ND-fed mice. At the phylum level, consistent with the previous study (Turnbaugh et al., 2006, 2009a), we observed a decreased abundance of Bacteroidetes and an increased ratio of F/B in HFD-fed mice. At the family level, Erysipelotrichaceae (phylum Firmicutes) was previously confirmed to be abundant in the gut microbiota after HFD feeding (Turnbaugh et al., 2009b; Zietak et al., 2016), which is similar to our results and the elevated Erysipelotrichaceae were restored by Api intervention. *Akkermansia* (family Akkermansiaceae) has been proposed to have many metabolic protective effects, such as decreasing body fat mass, adipose tissue inflammation, metabolic endotoxemia, and improving intestinal barrier integrity (Anhe et al., 2015; Dao et al., 2016). In this study, we found that there was a decreased abundance of *Akkermansia* in HFD-fed mice and that the abundance was restored by Api supplementation. Intriguingly, we found a significantly decreased abundance of Muribaculaceae in HFD-fed mice (*p* < 0.001), although Api supplementation did not restore its relative abundance. This finding indicates that decreased Muribaculaceae abundance may contribute to obesity-associated metabolic derangement and supports the view of Muribaculaceae as a biomarker of a healthy intestinal microbiome (Yang et al., 2019). In addition, our study revealed that the relative abundance of Eggerthellaceae was markedly increased in HFD-fed mice and that Api supplementation mildly restored its relative abundance, in contrast to a previous study, which reported that Eggerthellaceae was inversely correlated with body weight gain (Rodriguez-Daza et al., 2020). The Spearman correlation analysis exhibited that *Faecalibaculum*, *Dubosiella* (family Erysipelotrichaceae), and *Family_XIII_AD3011_group* (family Anaerovoracaceae) were strongly positively correlated with obesity, inflammation, intestinal permeability, and metabolic endotoxemia-related indexes, while *Incertae_Sedis* (family Ruminococcaceae) was negatively correlated with these indexes. This finding revealed that the former three bacteria may partly participate in the disruption of the intestinal barrier and facilitate LPS release into the blood, resulting in aggravation of metabolic dysfunction, while the last bacterium may prevent its progression. Bacteria within *Faecalibaculum*, *Dubosiella*, *Family_XIII_AD3011_group*, and *Incertae_Sedis* were significantly restored by Api supplementation in HFD-fed mice (*p* < 0.05). These results demonstrate that Api supplementation improves intestinal barrier integrity, metabolic disorders, and reshapes the composition of the gut microbiota by decreasing *Faecalibaculum* and *Dubosiella*, while increased *Akkermansia* and *Incertae_Sedis* may be partly responsible for Api’s beneficial effect. These observations suggest that Api may exert anti-obesity effects by changing the F/B ratio and regulating the levels of certain other bacterial species.

At the pathway level, there is a growing body of literature that demonstrates the Bacteroidetes are enriched for several carbohydrate metabolism pathways, whereas the Firmicutes are enriched for transport systems (Turnbaugh et al., 2009a). Our results exhibit that pathways for membrane transport and xenobiotics biodegradation and metabolism were simultaneously promoted in HFD-fed mice, including pathways for ABC transporters, the phosphotransferase system (PTS), benzoate degradation, and styrene degradation. In contrast, the pathways connected with amino acid metabolism, carbohydrate metabolism, energy metabolism, glycan biosynthesis and metabolism, and lipid metabolism were remarkably enriched in Api-supplemented mice, including pathways for oxidative phosphorylation, glycosaminoglycan degradation, lipopolysaccharide biosynthesis, and fatty acid biosynthesis. Therefore, compared to HFD feeding, we propose that Api supplementation can lead to an obvious transformation in the function of the intestinal biological community which is proved mainly by the increments in amino acid, carbohydrate, energy, glycan, and lipid metabolism.

However, the above results have only revealed the potential role of Api in modulating the gut microbiota thus further improve obesity-related metabolic syndrome. To further reinforce the relationship between, we transferred the gut microbiota from Api-treated obese mice to antibiotic-pretreated mice. Noteworthily, fecal transplants in Api receivers imitated the effects of orally administrated Api on the gut microbiota. Similar alterations of obesity-related indexes were observed in the FMT and Api receivers showed improved metabolic syndrome compared with HFD receivers. At the taxonomic level, in FMT, the alterations of the aforementioned bacteria were consistent with the Api-treatments study. These results further confirm the credibility that Api ameliorates obesity-related metabolic syndrome partially through the mediation of the gut microbiota.

Although encouraged by our findings, we admit some limitations of this work. For FMT, we transferred the feces from Api-treated obese mice to conventional HFD-fed receivers rather than germ-free recipient mice for some restrictions. Hence, the influence of the conventional mice’s gut microbiota could not be excluded. Moreover, our study only reveals that the potential mechanism of Api’s improvement of host metabolism by altered gut microbiota is through the prevention of LPS into systemic circulation, while Api’s production of microbiome-derived metabolites and their modulatory effects have not been taken into account. SCFAs, BCAAs, and BAs are microbiome-derived metabolites that have a significant influence on gut microbiota and host metabolism. Hence, these metabolites should be examined in future studies to illustrate the detailed mechanism of Api.

In conclusion, our studies substantiate that Api can mitigate metabolic syndrome by reshaping the gut microbiota structure and changing the F/B ratio. Our findings confirm that long-term oral administration of Api can be beneficial and provide a potential solution in therapeutic strategy targeted at the gut microbiota to overcome obesity and its complications.

## Data Availability Statement

The datasets presented in this study can be found in online repositories. The names of the repository/repositories and accession number(s) can be found at: https://www.ncbi.nlm.nih.gov/, PRJNA761909.

## Ethics Statement

The animal study was reviewed and approved by Animal Studies Committee of China-Japan Friendship Hospital.

## Author Contributions

LP and YQ conceived and designed this study and wrote and revised the manuscript. YQ, YZ, and XY helped to took samples and performed the experiments. YQ and ZZ analyzed the data. WZ, HL, and LG helped to perform the analysis with constructive discussions and modified the manuscript. All authors reviewed and approved the final manuscript.

## Funding

This work was supported by the National Natural Science Foundation of China (81970713 and 82170817), Beijing Municipal Natural Science Foundation of China (7182147), Capital’s Funds for Health Improvement and Research (2018-2-4062), and Joint Project of BRC-BC (Biomedical Translational Engineering Research Center of BUCT-CJFH, XK2020-10).

## Conflict of Interest

The authors declare that the research was conducted in the absence of any commercial or financial relationships that could be construed as a potential conflict of interest.

## Publisher’s Note

All claims expressed in this article are solely those of the authors and do not necessarily represent those of their affiliated organizations, or those of the publisher, the editors and the reviewers. Any product that may be evaluated in this article, or claim that may be made by its manufacturer, is not guaranteed or endorsed by the publisher.

## Supplementary Material

**Supplementary Figure S1**Apigenin reduces Epi-WAT weight in HFD-fed mice.

(A) Epi-WAT weight. (B) Epi-WAT weight/Body weight ratio. Error bars are expressed as means ± SEM (*n* = 6). Statistical significance was determined by one-way ANOVA with Tukey tests for multiple-group comparisons. ^*^*p* < 0.05, ^**^*p* < 0.01, and ^***^*p* < 0.001 vs. ND; ^#^p < 0.05 ^##^p < 0.01, and ^###^p < 0.001 vs. HFD.

## Abbreviations

AB-PAS: Alcian Blue Periodic Acid-Schiff
ALT: Alanine Aminotransferase
ANOSIM: Non-parametric Analysis of Similarities
Api: Apigenin
AST: Aspartate Aminotransferase
BAs: Bile Acids
BCAAs: Branched-Chain Amino Acids
CMC-Na: Sodium Carboxymethyl Cellulose
ELISA: Enzyme Linked Immunosorbent Assay
Epi-WAT: Epididymal White Adipose Tissue
F/B: Firmicutes/Bacteroidetes
FITC: Fluorescein Isothiocyanate
FMT: Fecal Microbiota Transplantation
GAPDH: Glyceraldehyde 3-Phosphate Dehydrogenase
GTT: Glucose Tolerance Test
HE: Hematoxylin And Eosin
HDL-c: High-Density Lipoprotein-Cholesterol
HFD: High-Fat Diet
IL-10: Interleukin 10
IL-1β: Interleukin 1β
ITT: Insulin Tolerance Tests
KEGG: Kyoto Encyclopedia of Genes and Genomes
LDL-c: Low-Density Lipoprotein-Cholesterol
LPS: Lipopolysaccharide
MCP-1: Monocyte Chemoattractant Protein-1
mRNA: Messenger RNA
NAFLD: Non-Alcoholic Fatty Liver Disease
ND: Normal Diet
ORO: Oil Red O
OTUs: Operational Taxonomic Units
PCoA: Principal Co-ordinates Analysis
PICRUSt2: Phylogenetic Investigation of Communities by Reconstruction of Unobserved States 2
PTS: Phosphotransferase System
QIIME: Quantitative Insights Into Microbial Ecology
qRT-PCR: Real-time Quantitative Reverse Transcription PCR
SCFAs: short-chain fatty acids
TC: Total Cholesterol
TG: Triglycerides
TLR4: Toll-Like Receptor 4
TNF-α: Tumor Necrosis Factor-α
ZO-1: Zonula Occludens-1
