# Supplementary material for: Apigenin Alleviates Obesity-Associated Metabolic Syndrome by Regulating the Composition of the Gut Microbiome
Source: Front Microbiol. 2022 Jan 3;12:805827. doi: 10.3389/fmicb.2021.805827 (PMC8762173; doi:10.3389/fmicb.2021.805827)
Supplement: Supplementary file 1 [file Table_1.docx]

**Table S1**

Primer Sequences

| **Genes** | **Primer sequence (5′-3′)** | **Product (bp)** | **GenBank accession** |
| --- | --- | --- | --- |
| *TNF-α* | F: TAGCCAGGAGGGAGAACAGA  R: TTTTCTGGAGGGAGATGTGG | 127 | NM_001278601.1 |
| *IL-1β* | F: TTGAAGAAGAGCCCATCCTC  R: CAGCTCATATGGGTCCGAC | 140 | XM_006498795.3 |
| *IL-10* | F: GCTCTTACTGACTGGCATGAG  R: CGCAGCTCTAGGAGCATGTG | 105 | NM_010548.2 |
| *MCP-1* | F: TCTGGGCCTGCTGTTCACA  R: GGATCATCTTGCTGGTGAATGA | 110 | NM_011333.3 |
| *ZO-1* | F: GCCGCTAAGAGCACAGCAA  R: GCCCTCCTTTTAACACATCAGA | 171 | NM_001163574 |
| *Occludin* | F: ATGTCCGGCCGATGCTCTC  R: TTTGGCTGCTCTTGGGTCTGTAT | 308 | NM_008756.2 |
| *GAPDH* | F: GCATCCACTGGTGCTGCC  R: TCATCATACTTGGCAGGTTTC | 145 | XM_001476707.5 |
