# Supplementary figures and images for: Apigenin Alleviates Obesity-Associated Metabolic Syndrome by Regulating the Composition of the Gut Microbiome
Source: Front Microbiol. 2022 Jan 3;12:805827. doi: 10.3389/fmicb.2021.805827 (PMC8762173; doi:10.3389/fmicb.2021.805827)

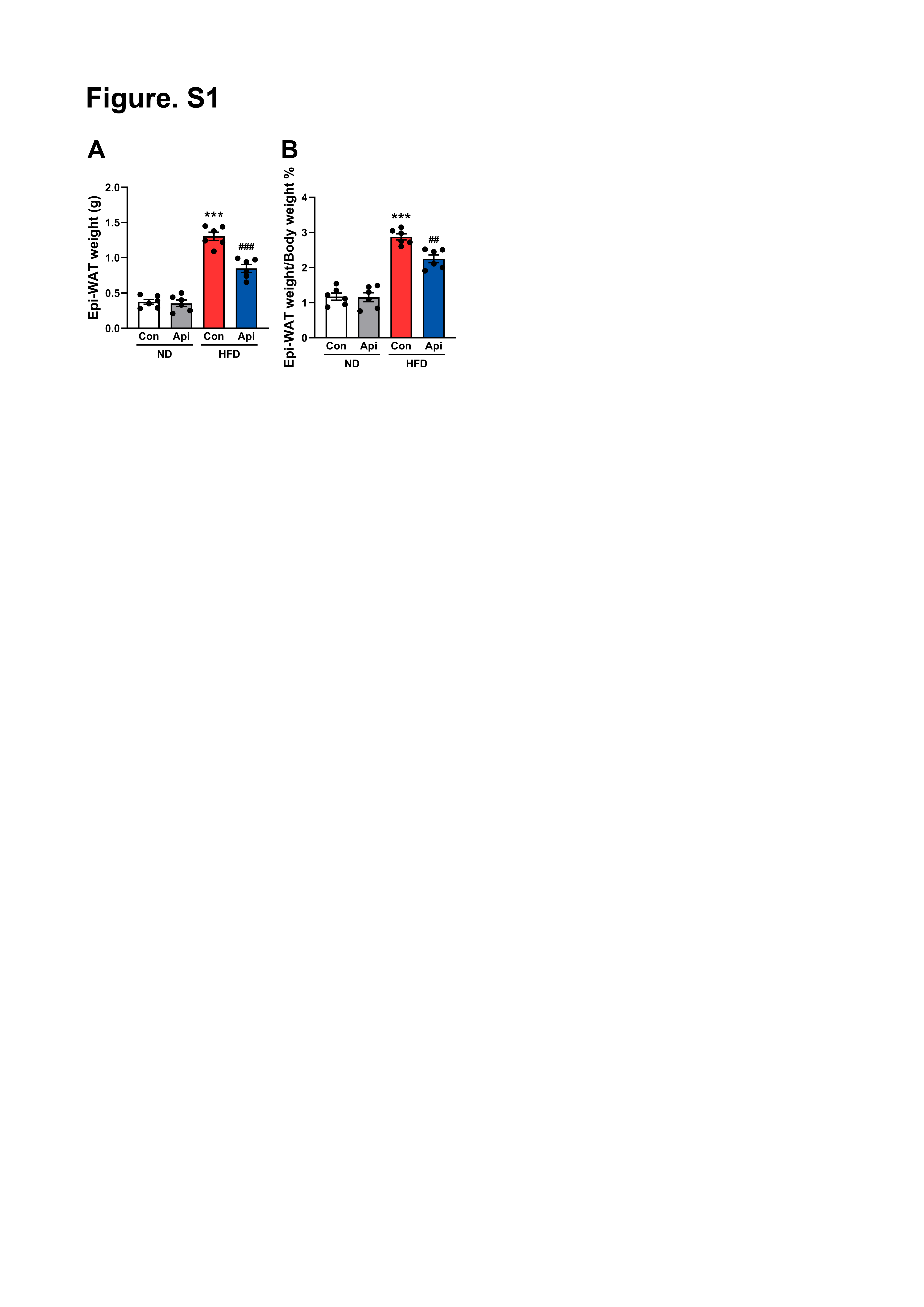

Supplement: Supplementary file 2 [file Image_1.tif]
